# Multiplex real-time reverse transcription recombinase-aided amplification assay for the detection of SARS-CoV-2, influenza A virus, and respiratory syncytial virus

**DOI:** 10.1128/spectrum.02759-24

**Published:** 2025-05-05

**Authors:** Yuandong Zhou, Chudan Liang, Zhenyu Long, Linjin Fan, Yulong Wang, Zequn Wang, Xiaofeng Yang, Pengfei Ye, Jingyan Lin, Wendi Shi, Huijun Yan, Linna Liu, Jun Qian

**Affiliations:** 1 Zhongshan School of Medicine, Sun Yat-sen University, Guangzhou, Guangdong, China; 2 Institute of Infectious Diseases, Guangzhou Eighth People’s Hospital, Guangzhou Medical University, Guangzhou, Guangdong, China; 3 School of Public Health (Shenzhen), Shenzhen Campus of Sun Yat-sen University, Shenzhen, Guangdong, China; 4 Shenzhen Key Laboratory of Pathogenic Microbes and Biosafety, Shenzhen, Guangdong, China; 5 Guangdong Provincial Highly Pathogenic Microorganism Science Data Center, Guangzhou, Guangdong, China; University of Chicago, Chicago, Illinois, USA

**Keywords:** RT-RAA, IAV, SARS-CoV-2, RSV, isothermal amplification, rapid diagnosis

## Abstract

Influenza A virus (IAV), severe acute respiratory syndrome coronavirus 2 (SARS-CoV-2), and respiratory syncytial virus (RSV) are significant pathogens that induce respiratory symptoms in humans, significantly impacting healthcare systems, economic activities, and human life on a global scale. The clinical manifestations associated with SARS-CoV-2, IAV, and RSV exhibit similarities. Cases of clinical coinfection are prevalent, highlighting the necessity for rapid differential diagnosis to avoid treatment delays. Traditional reverse transcription quantitative polymerase chain reaction (RT-qPCR) methods are often time-consuming and limited to central laboratories, necessitating the development of rapid and *in situ* screening techniques to accommodate broad and large-scale testing requirements. In light of the possibility of coinfection with multiple viral strains, a multiplex detection method known as triplex reverse transcription recombinase-aided amplification (RT-RAA) has been established to facilitate the simultaneous detection of these three pathogens. The triplex real-time RT-RAA assay operates at a constant temperature of 42°C and achieves detection within 30 minutes.The limits of detection (LOD) for IAV, SARS-CoV-2, and RSV were 138, 198, and 162 copies per reaction, respectively, comparable in order of magnitude to the 100 copies per reaction observed in the triplex RT-qPCR assay. Importantly, no cross-reactivity was observed with other significant viruses. In addition, compared with the triplex RT-qPCR assay, the triplex real-time RT-RAA assay demonstrated a high degree of concordance in the detection of IAV, SARS-CoV-2, and RSV in 58 clinical specimens. Overall, the triplex real-time RT-RAA assay holds considerable promise as a powerful tool for the rapid screening of respiratory diseases, owing to its advantages, including expedited detection, ease of operation, strong specificity, and high sensitivity.

**IMPORTANCE:**

It is well established that SARS-CoV-2, IAV, and RSV are significant pathogens that elicit respiratory symptoms in humans. In this study, we successfully developed a triplex real-time RT-RAA assay for the simultaneous rapid detection of SARS-CoV-2, IAV, and RSV. This assay is distinguished by its high specificity, sensitivity, and user-friendliness. It addresses the limitations associated with the necessity of specialized biological laboratories and the time-consuming, complex procedures that impede rapid diagnosis. This assay holds significant importance for the early diagnosis and epidemiological surveillance of respiratory tract infections, providing comprehensive and timely diagnostic information for clinical practice. Furthermore, this assay serves as a valuable tool for facilitating extensive and large-scale screening of SARS-CoV-2, IAV, and RSV.

## INTRODUCTION

Severe acute respiratory syndrome coronavirus-2 (SARS-CoV-2) has precipitated the global coronavirus disease 2019 (COVID-19) pandemic (1), resulting in more than 771 million confirmed cases and approximately 6.9 million fatalities worldwide as of September 4, 2023, according to a real-time COVID-19 epidemic report from the World Health Organization (WHO) (2). This pandemic has imposed significant economic and social burdens across the globe (3). SARS-CoV-2 is classified as a β-coronavirus and is characterized as a positive-sense single-stranded RNA (+ssRNA) virus. By contrast, the influenza A virus (IAV) is a highly contagious respiratory pathogen that belongs to the Orthomyxoviridae family of segmented, negative-sense single-stranded RNA viruses, which are known to facilitate seasonal influenza outbreaks and pose substantial threats to global public health (4). IAV has historically jeopardized both the global livestock industry and human health, garnering considerable attention from the scientific community (5). In addition, respiratory syncytial virus (RSV) is an enveloped, filamentous, negative-strand RNA virus responsible for significant respiratory illnesses worldwide. RSV is recognized as one of the leading causes of hospitalization among infants, elderly individuals, residents of long-term care facilities, and other vulnerable populations (6). In a study involving 6,965 patients diagnosed with SARS-CoV-2, tests for respiratory viral coinfections revealed that 583 (8.4%) patients were coinfected with other viruses: 227 patients had influenza viruses, 220 patients were infected with respiratory syncytial virus, and 136 patients had adenoviruses (7). The clinical manifestations associated with SARS-CoV-2, IAV, and RSV exhibit considerable overlap, primarily presenting respiratory symptoms, including fever, cough, dyspnea, and fatigue (8, 9). Given the potential for coinfection and the manifestation of analogous clinical symptoms, a differential diagnosis assay is essential.

The effective differentiation of these viruses from SARS-CoV-2 and other respiratory viruses necessitates the use of specific and sensitive assays to facilitate precise treatment, prevention, and control measures. Reverse transcription quantitative polymerase chain reaction (RT-qPCR) is a conventional technique employed for the detection of SARS-CoV-2, IAV, and RSV (10). However, practical applications reveal several limitations associated with this method, including cumbersome operational procedures, high equipment requirements, and extended processing times. These factors restrict its applicability in resource-limited settings and point-of-care testing (POCT). Consequently, there is a pressing need to develop a rapid, straightforward, cost-effective, and sensitive diagnostic method suitable for field use.

Isothermal nucleic acid amplification assays do not require a thermal cycler, thus simplifying the operation process. This technique has attracted significant attention in recent years and has led to the development of rapid and POC detection methods as alternatives to regular PCR. Among these isothermal assays, recombinase-aided amplification (RAA) is a remarkably appealing isothermal amplification technique that reacts at a much lower temperature (37–42°C) and in a much shorter time (usually within 30 min). RAA isothermal amplification relies on the participation of many enzymes and proteins. There are three key enzymes in the system: single-stranded DNA-binding protein (SSB), recombinase, and strand-displacing DNA polymerase. In the RAA reaction, the recombinant enzyme protein forms a complex with the primer and searches for homologous sequences in double-stranded DNA. The primers are then inserted into homologous sites through the chain replacement activity of the recombinase, and the single-stranded binding protein stably replaces the DNA strand. The recombinant enzyme is then broken down so that the 3′ ends of the primer is chain replaced and bound to the polymerase, allowing it to lengthen the primer, exponentially expanding the target region on the template by repeating the process cyclically (11). Another technology similar to RAA is RPA (Recombinase Polymerase Amplification). The key difference is that RAA utilizes a recombinant enzyme derived from bacteria or fungi, whereas RPA relies on a phage recombinant enzyme, which is more challenging to obtain. The amplification processes for both methods are identical, achieving *in vitro* DNA amplification through binding, strand displacement, and extension (12). In the RAA system, the addition of specific fluorescent probes can monitor DNA amplicons in real time. If reverse transcriptase is combined with a specific fluorescent probe, real-time monitoring of RNA amplicons can be realized (13). RAA has the characteristics of high sensitivity and specificity, a short reaction time, simple operation, intuitive judgment of results, and suitability for rapid diagnosis in the field. At present, this method has been successfully applied in the detection of viruses, bacteria, and other pathogens (14).

Many RAA and qPCR assays have been established for the effective identification and differentiation of influenza viruses and other respiratory pathogens (15). Consequently, the authors developed a triplex real-time RT-RAA assay that is capable of simultaneously detecting and differentiating among SARS-CoV-2, IAV, and RSV within a single tube. This assay utilizes various clinical specimens obtained from the respiratory tract, including nasopharyngeal swabs, oropharyngeal swabs, and sputum.

## MATERIALS AND METHODS

### Virus and clinical samples

A total of 58 samples, comprising IAV, SARS-CoV-2, and RSV, were utilized in this study. These samples were collected between 2023 and 2024 and included 43 primary clinical specimens obtained via oropharyngeal swabs and alveolar lavage fluid, as well as 15 viruses isolated through cell culture. All samples included in this research were sourced from the Guangzhou Eighth People’s Hospital. Detailed information regarding all clinical samples is shown in Table S1.

### Design of primers and probes for the triplex real-time RT-RAA assay

**Fig 1:**
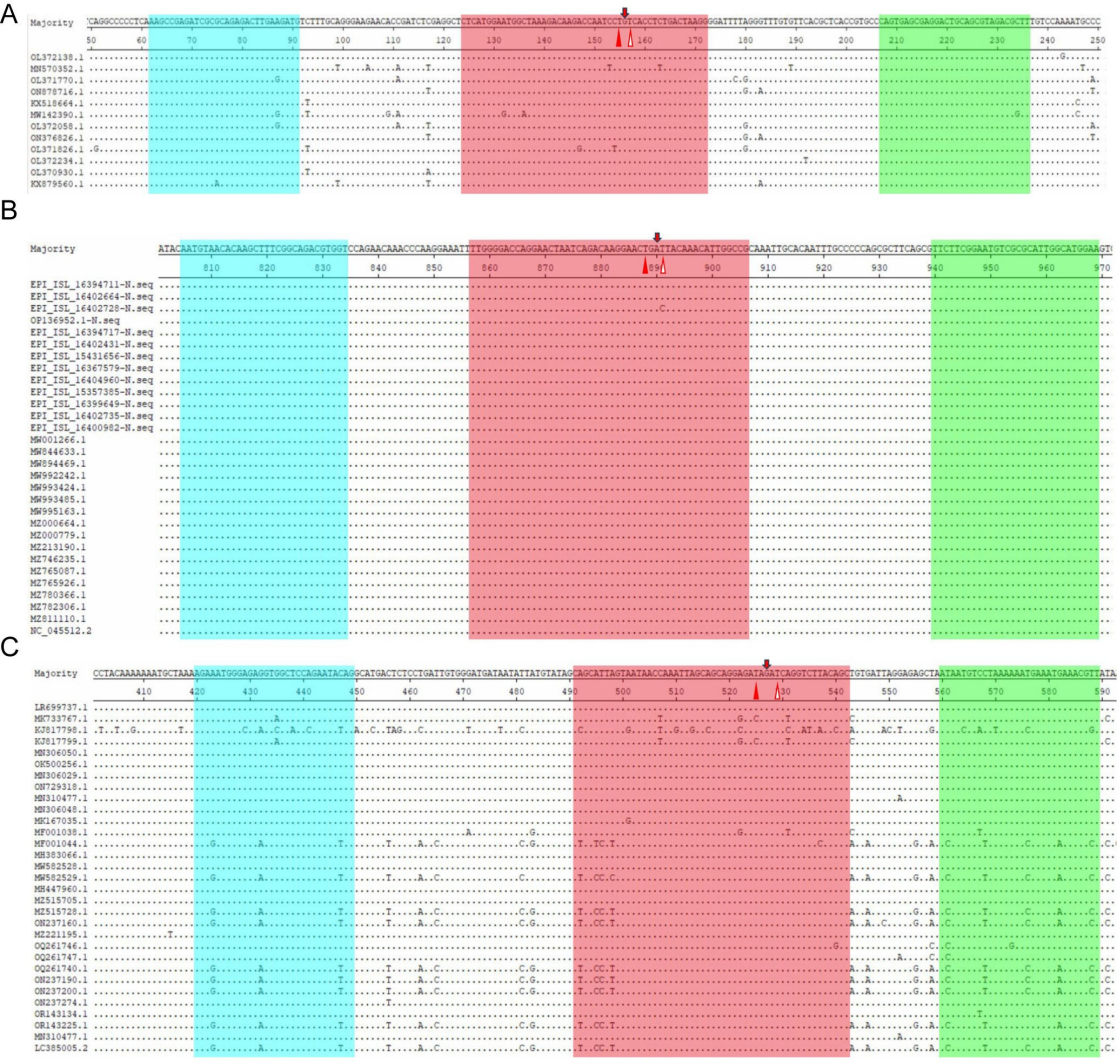
Locations of the triplex real-time RT-RAA primers and probe on the gene sequence of the target gene of the pathogen. Utilize dots to denote nucleotide residues that correspond to the majority. The forward primer is indicated in blue, the reverse primer in green, and the exo probe in red. The two T residues within the exo probe, which are labeled with a fluorophore and a quencher, are represented by solid and hollow triangles, respectively. The THF is indicated by an arrow. (**A**) Illustration of the position of various IAV strains on the M gene sequence; (**B**) the position of different SARS-CoV-2 strains on the N gene sequence; and (**C**) the position of various RSV strains on the N gene sequence.

The M gene sequences from 12 distinct strains of IAV, the N gene sequences from 30 different strains of SARS-CoV-2, and the N gene sequences from 31 different strains of RSV were aligned utilizing DNASTAR software. In addition, SnapGene software was employed for the design of primers and probes. The design of RAA primers adhered to several fundamental principles: the primer length should be at least 30 base pairs (bp), ideally ranging from 30 to 36 bp; the length of the amplicon should not exceed 500 bp, with a preferred range of 100 to 200 bp; the GC content should be greater than 20% and less than 70%; the melting temperature (Tm) of the primers should be greater than 50°C and less than 100°C; The base at the 3′ end of the primer should be highly conserved; it is advisable to avoid short sequences with numerous repeats in the primer; the maximum length of a mononucleotide repeat should not exceed 5; and primer sequences that can form hairpin structures or dimers should be avoided. Primers and probes were designed and screened in accordance with the guidelines provided by TwistDx (16). The final primers and probes for the triplex real-time RT-RAA amplification developed in this study are presented in Fig. 1 and Table 1.

**TABLE 1:** The primers and probes utilized in the triplex real-time RT-RAA assay^*a*^

Virus	Name	Sequences (5′→3′)	Gene	Product
IAV	F62-91	AAGCCGAGATCGCGCAGAGACTTGAAGATG	M	175 bp
	R207-236	AAGCGTCTACGCTGCAGTCCTCGCTCACTG		
	P124-172	CTCATGGAATGGCTAAAGACAAGACCAATCC(FAM-dT)(THF)(BHQ1-dT)CACCTCTGACTAAGG(C3-spacer)		
SARS-CoV-2	F805-834	AATGTAACACAAGCTTTCGGCAGACGTGGT	N	165 bp
	R940-969	TTCCATGCCAATGCGCGACATTCCGAAGAA		
	P857-906	TTGGGGACCAGGAACTAATCAGACAAGGAAC(TAMRA-dT)G(THF)(BHQ2-dT) TACAAACATTGGCCG(C3-spacer)		
RSV	F420-449	AGAAATGGGAGAGGTGGCTCCAGAATACAG	N	170 bp
	R559-589	AACGTTTCATTTCATTTTTTAGGACATTATT		
	R491-542	CAGCATTAGTAATAACCAAATTAGCAGCAGGAGA(ROX-dT)A(THF)A(BHQ2-dT)CAGGTCTTACAGC(C3-spacer)		

^*a*^ FAM-dT refers to a thymidine nucleotide that is conjugated with fluorescein; TAMRA-dT denotes a thymidine nucleotide that is conjugated with tetramethyl-6-carboxyrhodamine; ROX-dT indicates a thymidine nucleotide that is conjugated with Rhodamine X; BHQ1-dT represents a thymidine nucleotide that is conjugated with black hole quencher 1; BHQ2-dT signifies a thymidine nucleotide that is conjugated with black hole quencher 2. THF stands for tetrahydrofuran spacer, while C3-Spacer refers to a C3 spacer located at the 3' end to inhibit elongation.

### Screening for optimal primers in a triplex real-time RT-RAA assay

**Fig 2:**
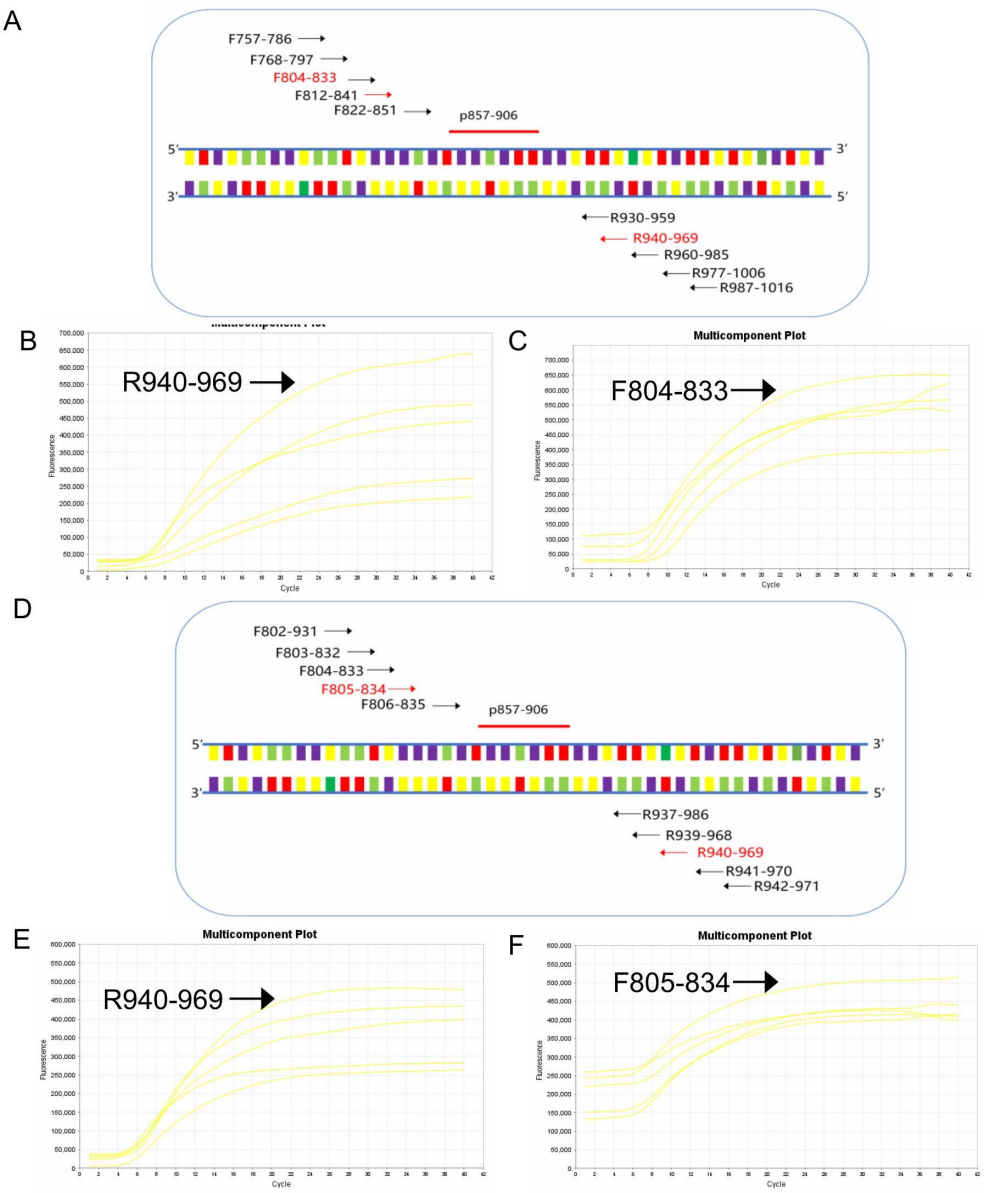
Screening of optical primers for real-time RT-RAA detection of SARS-CoV-2. (**A**) Schematic representation of the primary primer screening process. In the primer nomenclature, the numerical values denote the specific positions within the N gene of SARS-CoV-2 (GenBank accession no. MW001266.1). (**B**) Results of the primary reverse primer screening. The forward primer F768-797 was randomly selected to evaluate all five reverse primers. (**C**) Results of the primary forward primer screening. The selected reverse primer R940-969 was utilized to assess all five forward primers. (**D**) Schematic representation of the secondary primer screening process. (**E**) Results of the secondary reverse primer screening. The selected forward primer F805-834 was employed to evaluate all seven reverse primers. (**F**) Results of the secondary forward primer screening. The selected reverse primer R940-969 was utilized to assess all seven forward primers.

**Fig 3:**
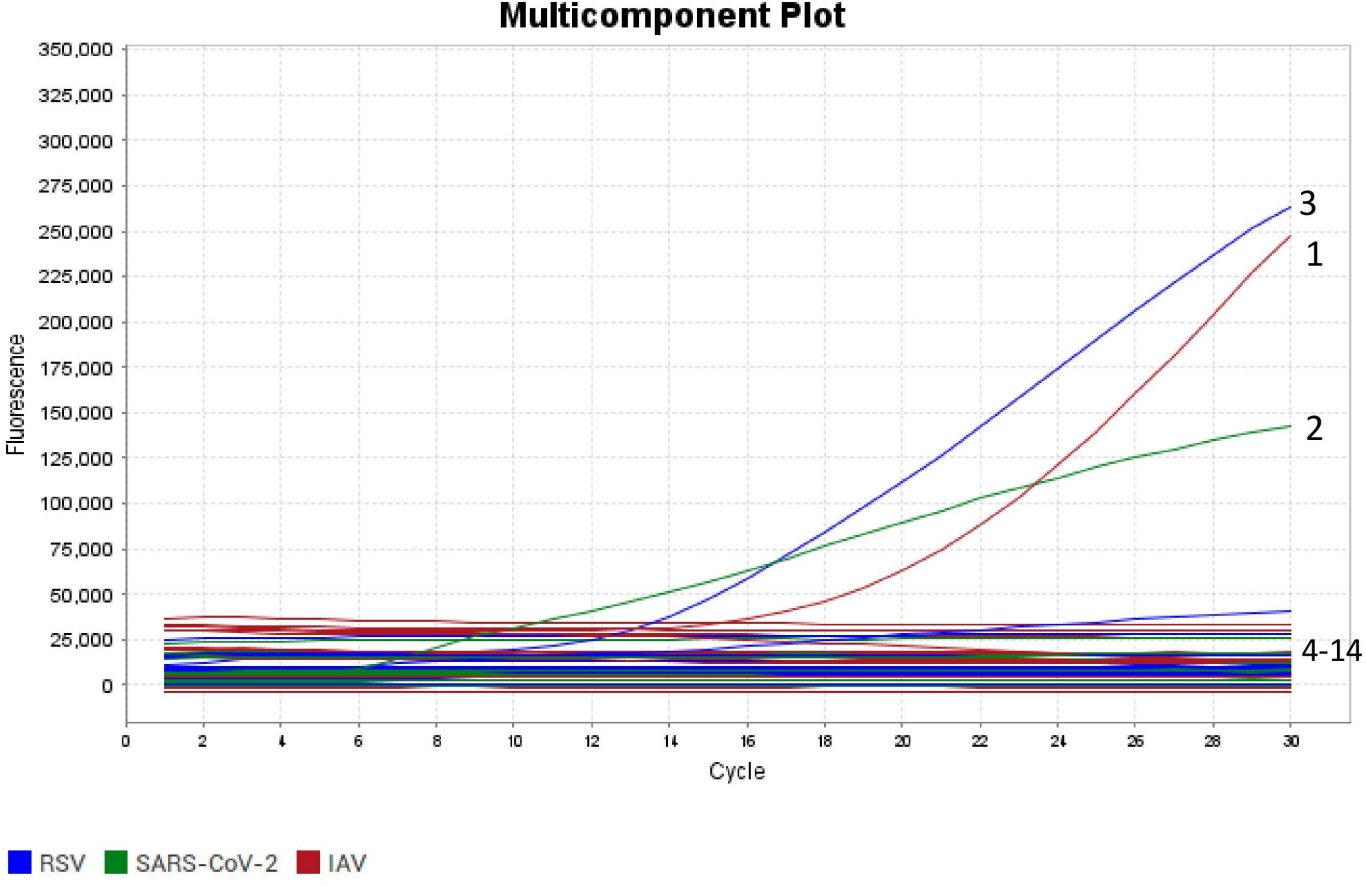
Specificity of the triplex real-time RT-RAA assay. The curves labeled 1 to 14 correspond to nucleic acid templates for the following pathogens: IAV, SARS-CoV-2, RSV, HPIV1, HPIV3, HMPV, HAdV, HRV, HCoV, C. pneumonia, M. pneumonia, IBV, HBoV1, along with a negative control.

**Fig 4:**
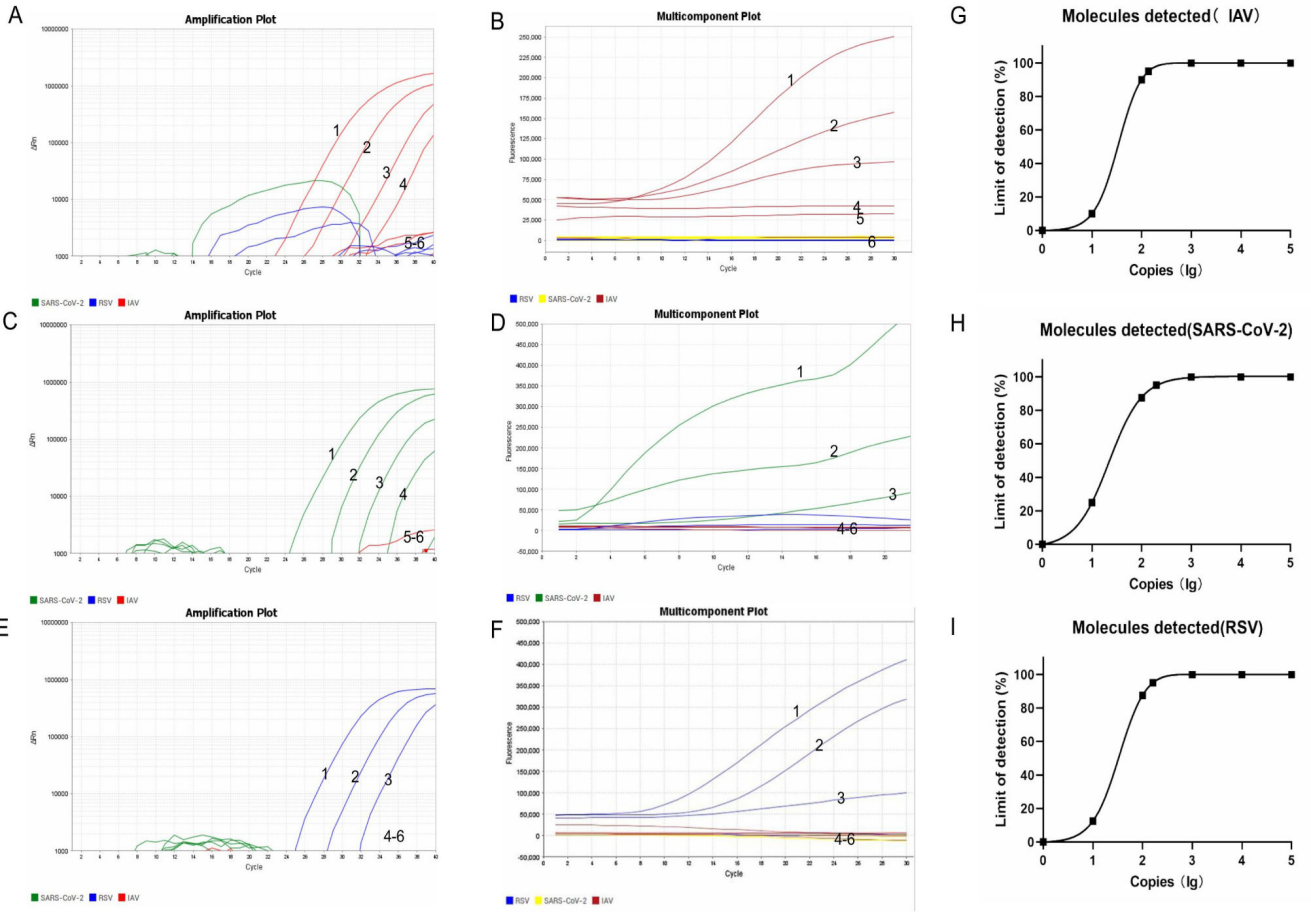
Sensitivity of the triplex real-time RT-RAA assay in comparison to the triplex RT-qPCR. Sensitivity of the triplex real-time RT-RAA assay in comparison to the triplex RT-qPCR. Curves 1–6 correspond to 1× (10)^4^ to 1 × (10)^0^ copies and the negative control, respectively. (**A**) Sensitivity analysis of IAV detection using the triplex RT-qPCR assay. (**B**) Sensitivity analysis of IAV detection using the triplex real-time RT-RAA assay assay. (**C**) Sensitivity analysis of SARS-CoV-2 detection using the triplex RT-qPCR assay. (**D**) Sensitivity analysis of SARS-CoV-2 detection using thetriplex real-time RT-RAA assay. (**E**) Sensitivity analysis of RSV detection using the triplex RT-qPCR assay. (**F**) Sensitivity analysis of RSV detection using the triplex real-time RT-RAA assay. (**G–I**) The detection limits of the triplex real-time RT-RAA assay, at a 95% probability, were 138, 198, and 162 copies per reaction for IAV, SARS-CoV-2, and RSV, respectively.

Initially, we identified an optimal probe for the conserved region of the gene, as illustrated in Table 2. Subsequently, we designed five forward primers and five reverse primers surrounding the probe region. The strategies employed for primer screening are outlined in the TwistDx guidelines (16). In summary, we screened all five reverse primers using a randomly selected forward primer, identified the most effective reverse primer, and subsequently utilized it to screen all forward primers, thereby determining a sensitive primer pair. To enhance the sensitivity of the primer combination, a second round of primer screening was conducted following the same methodology. In conclusion, similar two-round primer screening procedures were applied to SARS-CoV-2, IAV, and RSV, as represented in Fig. 2 to 4.

**TABLE 2:** A comparative analysis of the triplex real-time RT-RAA method and the triplex RT-qPCR assay using clinical samples

Virus	Triplex real-time RT-RAA	Triplex RT-qPCR	Sample type	Sensitivity	Specificity	Kappa
SARS-CoV-2	28	28	Oropharyngeal swabs	100%	100%	1
IAV	18	18	Oropharyngeal swabs and cultured isolates			
RSV	6	6	Oropharyngeal swabs and alveolar lavage fluid			
Negative result	6	6	Oropharyngeal swabs, alveolar lavage fluid, and cultured isolates			

### Nucleic acid extraction

Total RNA was extracted from each sample utilizing TRIzol reagent (Magen, Guangzhou, China) in accordance with the manufacturer’s instructions. The extracted RNA was subsequently eluted into 30 µL of nuclease-free water and stored at −80°C for future applications.

### Triplex real-time RT-RAA assay

The assays were conducted utilizing a kit (#S002ZC) from Hangzhou ZC Bio-Sci & Tech Co., Ltd. (China). The primers and probes employed in the study are detailed in Table 1. In summary, the triplex real-time RT-RAA system (50 µL per reaction) comprises the following components: buffer A (25 µL); three forward primers (10 µM), each at 1.34 µL, totaling 4.0 µL; three reverse primers (10 µM), each at 1.34 µL, also totaling 4.0 µL; three exo probes (10 µM), each at 0.6 µL, summing to 1.8 µL; nuclease-free water (8.7 µL); nucleic acid template (4.0 µL); and buffer B (2.5 µL). The reaction tubes were subsequently placed in the QuantStudio real-time PCR System (Applied Biosystems, America) and incubated at 42°C for 30 minutes (1 cycle/min) to monitor the fluorescence signal in real time.

### Triplex RT-qPCR assay

The assays were conducted utilizing a kit (BNCC378336) obtained from the BeNa Culture Collection in China. The 25 µL Triplex RT-qPCR system comprised the following components: 23 µL of TaqPath One-Step Multiplex Master Mix and 2 µL of nucleic acid template. The reaction tubes were subsequently placed in the QuantStudio Real-Time PCR System (Applied Biosystems) under the following conditions: an initial step at 95°C for 3 minutes, followed by 40 cycles of 94°C for 10 seconds and 56°C for 30 seconds.

### Analytical specificity

The specificity of the triplex real-time RT-RAA assay was assessed by utilizing a range of significant pathogens, including IAV, SARS-CoV-2, RSV, Human Parainfluenza Virus 1 (HPIV1), Human Parainfluenza Virus 3 (HPIV3), Human Metapneumovirus (HMPV), Human Adenovirus (HAdV), Human Rhinovirus (HRV), Human Coronavirus (HCoV), Chlamydia pneumoniae (C. pneumoniae), Mycoplasma pneumoniae (M. pneumoniae), Influenza B virus (IBV), and Human Bocavirus 1 (HBoV1).

### Analytical sensitivity

After diluting the IAV-M plasmid (pUC57-IAV-M), the SARS-CoV-2-N plasmid (pUC57-SARS-CoV-2-N), and the RSV-N plasmid (pUC57-RSV-N), which were synthesized by RuiBiotech (China), by a factor of 10, plasmid concentrations ranging from 1 × 10^4^ to 1 × 10°Copies per μL were achieved. Four microliter of each dilution was utilized as a template to evaluate the sensitivity of the triplex real-time RT-RAA assay. For comparative purposes, the same template was concurrently tested using the triplex RT-qPCR assay. For a more accurate analysis of the limit of detection, 10 independent runs were conducted for the triplex real-time RT-RAA assay using a dilution series (10^5^–10°Copies per reaction) as templates. Probit regression analysis was performed on the data using IBM’s Statistical Product and Service Solutions (SPSS) software. The DNA copy number was calculated using the following formula: DNA copy number (copies/μL) = [plasmid concentration (ng/μL) ×10^−9^ × 6.02 ×10^23^]/[DNA length (nt) ×660].

### Assay performance with clinical specimens

We conducted an evaluation of the clinical performance of the triplex RT-RAA assay utilizing 58 respiratory specimens, which included throat swabs and viruses isolated through cell culture. Total nucleic acids were extracted from 30 µL of each clinical specimen employing TRIzol reagent (Magen, Guangzhou, China). The specimens were subsequently analyzed using the triplex real-time RT-RAA assay. For comparative purposes, the same template was concurrently tested using the triplex RT-qPCR assay.

### Statistical analysis

The probit regression analysis was performed at the 95% probability level to determine the detection limits. The kappa and *P* values for the triplex real-time RT-RAA and triplex RT-qPCR assays were computed. Data analysis was conducted using IBM’s Statistical Package for the Social Sciences (SPSS) software.

## RESULTS

### Localization of conserved gene amplicon targets in SARS-CoV-2, IAV, and RSV genomes

The nucleotide sequences of the N gene from 30 distinct strains of SARS-CoV-2 were aligned via DNASTAR software. SnapGene software was subsequently employed to design probes and primers targeting conserved regions within the N gene. Notably, the two modified thymine (T) residues in the selected probe (p857-906) are fully conserved across the 30 representative SARS-CoV-2 strains (Fig. 1B). Dots are employed to denote nucleotide residues that match the majority of nucleotide residues. The optimal primer pair, F805-834/R940-969, was identified through two rounds of primer screening. The forward primer (F805-834) is highlighted in blue, the reverse primer (R940-969) is indicated in green, and the exo probe (p857-906) is marked in red. The two T residues in the probe p857-906 are designated with a solid triangle and a hollow triangle, which are associated with a fluorophore (TAMRA) and a quencher (BHQ2), respectively (Fig. 1B).

The DNASTAR software was used to align the N gene sequences of 31 distinct strains of RSV and the M gene sequences of 12 different strains of IAV. In the case of IAV, the forward primer (F62-91) is indicated in blue, the reverse primer (R207-236) is represented in green, and the exo probe (p124-172) is depicted in red. The two T residues in the p124-172 probe are marked with a solid triangle and a hollow triangle, corresponding to a fluorophore (FAM) and a quencher (BHQ1), respectively (Fig. 1A). For RSV, the forward primer (F420-449) is shaded blue, the reverse primer (R559-589) is shaded green, and the exo probe (p491-542) is shaded red. Similarly, the two T residues in the p491-542 probe are labeled with a solid triangle and a hollow triangle, associated with a fluorophore (ROX) and a quencher (BHQ2), respectively (Fig. 1C).

### Screening primers

In the study of SARS-CoV-2, we initially identified an optimal probe, designated p857-906 (Fig. 2). Next, we developed five candidate forward primers (F757-786, F768-797, F804-833, F812-841, and F822-851) and five candidate reverse primers (R930-959, R940-969, R960-985, R977-1006, and R987) surrounding p857-906 (Fig. 2A). The detailed primer screening strategies are outlined in the TwistDx Guide. In summary, we conducted a screening of five reverse primers using the randomly selected forward primer F768-797, which revealed that the reverse primer R940-969 exhibited the most effective amplification effect (Fig. 2B). Subsequently, we screened five forward primers in conjunction with R940-969, identifying F804-833 as the most effective (Fig. 2C). Consequently, the primer pair F804-833/R940-969 demonstrated optimal performance in the initial round of screening. To further enhance the selection of primer pairs, we designed five new forward primers and five new reverse primers in proximity to F804-833 and R940-969, respectively (Fig. 2D). All five reverse primers were evaluated using F804-833, with R940-969 again showing the best amplification results (Fig. 2E). Subsequently, R940-969 was employed to screen all five forward primers, among which F805-834 yielded the most favorable results (Fig. 2F). Ultimately, after two rounds of primer screening, the optimal primer pair F805-834/R940-969 was selected.

In the case of IAV, we initially identified an optimal probe (p124-172). Following this approach, after two rounds of primer screening, we selected the optimal primer pair F62-91/R207-236 (Fig. S1). Similarly, for RSV, we identified an ideal probe (p491-542). Utilizing the same methodology, after two rounds of primer screening, we determined the optimal primer pair F420-449/R559-589 (Fig. S2). The final primers and probes for the triplex real-time RT-RAA amplification developed in this study are presented in Table 1.

### Analytical specificity

The results of the specificity analysis indicated that the triplex real-time RT-RAA assay yielded positive results for SARS-CoV-2, IAV, and RSV. By contrast, HPIV1, HPIV3, HMPV, HAdV, HRV, HCoV, *C. pneumoniae*, *M. pneumoniae*, IBV, and HBoV1 tested negative (Fig. 3). These findings suggest that the triplex real-time RT-RAA assay is specific for the detection of SARS-CoV-2, IAV, and RSV. For the specificity and sensitivity analyses, the triplex real-time RT-RAA assay required 30 minutes, whereas the triplex RT-qPCR assay required 1 hour.

### Analytical sensitivity

The sensitivity analysis of the triplex real-time RT-RAA assay was conducted using serial dilutions of the pUC57 plasmid, ranging from 1 × 10^4^ to 1 × 10^0^ copies per reaction, as templates. Each sample was tested in triplicate. The limits of detection for the triplex real-time RT-RAA assay targeting IAV, SARS-CoV-2 and RSV were tentatively determined to be 1 × 10^2^ copies per reaction (Fig. 4B, D, and F). For comparative analysis, the same template was concurrently evaluated using the triplex RT-qPCR assay. The detection limits for SARS-CoV-2, IAV, and RSV were determined to be 1 × 10^2^ copies per reaction (Fig. 4A, C, and E). Probit regression analyses further indicated that the detection limits of the triplex real-time RT-RAA assay, at a 95% probability, were 138, 198, and 162 copies per reaction for IAV, SARS-CoV-2, and RSV, respectively (Fig. 4G through I).

### Validation of triplex real-time RT-RAA assay in clinical specimens

Fifty-eight clinical samples, including alveolar lavage fluid, oropharyngeal swabs, and culture-isolated viruses, were subjected to evaluation using triplex real-time RT-RAA and triplex RT-qPCR assays. As presented in Table 2, in comparison with the RT-qPCR assay (Table S1), the real-time RT-RAA assay also demonstrated a sensitivity and specificity of 100%. The two assays exhibited a strong correlation, as indicated by a Kappa value of 1 (*P* < 0.001, Table 2). Furthermore, both the positive predictive value (PPV) and the negative predictive value (NPV) were found to be 100%. These pilot clinical results demonstrated that this triplex real-time RT-RAA assay has potential efficacy in clinical practice.

## DISCUSSION

The epidemics of SARS-CoV-2, IAV, and RSV have significantly impacted the global economy, public health, and human life. The rapid and reliable detection of these viruses is essential for controlling the spread of disease. Currently, RT-qPCR amplification serves as the primary method for detecting these viruses in China and other countries, playing a crucial role in the control and prevention of their transmission. Despite the high specificity and sensitivity associated with RT-qPCR testing, the occurrence of false-negative results in symptomatic patients and/or those with positive computed tomography (CT) scans presents a persistent challenge. Furthermore, the method is time-consuming and requires complex and costly equipment, as well as trained personnel (9). These limitations render RT-qPCR unsuitable for widespread application in field settings. Consequently, there is a pressing need to develop a faster, portable, and reliable diagnostic method for the amplification of these viruses.

Among the various molecular diagnostic methods, RAA has garnered significant public interest in recent years because of its distinctive characteristics. These include the ability to react at low and constant temperatures, the absence of the need for thermal cyclers, and the availability of multiple downstream detection formats. Other isothermal amplification technologies, such as loop-mediated isothermal amplification (LAMP), operate at temperatures ranging from 60°C to 65°C. Conventional LAMP methods typically require 15 to 60 minutes to achieve detection. LAMP amplification at elevated temperatures necessitates strict measures to prevent cross-contamination (17). Furthermore, LAMP is highly sensitive and susceptible to false positives. In recent years, many LAMP assays have become significantly faster when optimized with loop primers. For example, the ID Now assay, developed by Abbott Laboratories, utilizes instrumental and nicking endonuclease amplification (NEAR) technology to provide ultra-fast amplification of DNA or RNA. When combined with fluorescence detection, this method can yield results in 5 to 15 minutes or less, with positive results available within 5 minutes and negative results within 15 minutes (18). By contrast, RAA technology significantly reduces the detection time to between 10 and 30 minutes. RAA holds considerable promise as a cost-effective POC tool for extensive pathogen screening, particularly in medical-resource-limited settings. Recombinase polymerase amplification (RPA) is an isothermal nucleic acid amplification technique that rapidly detects target DNA/RNA at low temperatures using recombinase-primer complexes and strand-displacing polymerase, while PRA is another commonly used isothermal method similar to RAA.The key difference is that RAA utilizes a recombinant enzyme derived from bacteria or fungi, whereas RPA relies on a phage recombinant enzyme, which is more challenging to obtain. Therefore, the sensitivity and specificity of RAA and PRA are essentially equivalent; however, the cost of RPA is more expensive. Compared to other POC methods, such as molecular diagnostics and serological diagnostics, the advantages of RAA are evident in its effective amplification, straightforward operational procedures, and affordability (19).

The mutation and recombination of viral genomes present significant challenges for the detection of these viruses. In this study, we developed a triplex real-time RT-RAA assay for the rapid detection of these viral pathogens. During the primer and probe design phase, we aligned the conserved gene sequences of several representative strains. Initially, we selected an optimal exo probe, wherein the two T residues within the exo probe, labeled with fluorophores (FAM, TAMRA, ROX) and quenchers (BHQ1, BHQ2), exhibited 100% sequence homology with all representative strains. Subsequently, the RAA primers were designed on the basis of the TwistDx Guidelines (16).

In summary, we conducted a screening of all reverse primers using a randomly selected forward primer, subsequently identifying the most effective reverse primer for further screening against all the forward primers. Following the secondary screening of primers, the optimal primers were selected. The amplification threshold for the triplex real-time RT-RAA assay was determined to be 1 × 10^2^ copies of the recombinant plasmid per reaction, which is comparable to the threshold observed in triplex RT-qPCR (1 × 10^2^ copies per reaction). Probit regression analyses further indicated that the detection limits of the triplex real-time RT-RAA assay, at a 95% probability, were 138, 198, and 162 copies per reaction for IAV, SARS-CoV-2, and RSV, respectively. Among the 58 clinical tissue samples analyzed, the sensitivity and specificity of the real-time RT-RAA assay were both found to be 100% (58/58). These findings indicate that there was complete concordance between the two methodologies, with an overall agreement of 100%.

The RAA system incorporates reverse transcriptase, enabling the reverse transcription reaction to occur concurrently with the amplification process. This allows for the direct amplification of RNA molecules to be completed within 30 minutes at 42°C. By contrast, RT-qPCR requires a minimum of 1 h to achieve amplification. Furthermore, real-time RT-RAA amplification can be integrated with lateral flow assays to facilitate the visualization of detection results (20). In addition, this amplification method can be combined with microfluidic chip technology to enable multi-pathogen detection (21). Numerous studies have demonstrated that RAA technology, when paired with CRISPR systems, can achieve ultrahigh sensitivity and specificity for the detection of individual DNA or RNA molecules (22). Given its specificity, sensitivity, rapidity, and ease of operation, RAA technology holds significant promise for applications in the early diagnosis of animal diseases, rapid detection, and import-export quarantine, among other areas. In addition, we conducted a comparison to assess the impact of RNA extraction on detection methods. Positive samples confirmed to be influenza A virus were collected and utilized in the subsequent experiments. The sample with a high viral load (Ct=25.29) exhibited detectable yet less efficient amplification in the absence of RNA extraction, whereas the sample with a low viral load (Ct=33.65) necessitated RNA extraction for unequivocal detection. The results demonstrated that the lack of RNA extraction led to a substantial decrease in sensitivity. For future applications in POCT, optimizing alternative, more accessible methods for sample preparation is essential and will be the focus of our ongoing research.

In conclusion, a triplex real-time RT-RAA assay has been successfully developed for the detection of SARS-CoV-2, IAV, and RSV. This assay demonstrates the capability to detect these viruses rapidly, efficiently, and reliably. This assay holds significant importance for the early diagnosis and epidemiological surveillance of respiratory tract infections, providing comprehensive and timely diagnostic information for clinical practice. The portability and ease of use of the equipment render the RAA assay a promising POCT tool in medical-resource-limited settings, facilitating extensive and large-scale screening for SARS-CoV-2, IAV, and RSV.

## SUPPLEMENTAL MATERIAL

Supplemental figures spectrum.02759-24-s0001.docx Fig. S1 and S2.

Table S1 spectrum.02759-24-s0002.docx Specific information on clinical samples.

ASM does not own the copyrights to Supplemental Material that may be linked to, or accessed through, an article. The authors have granted ASM a non-exclusive, world-wide license to publish the Supplemental Material files. Please contact the corresponding author directly for reuse.
