# Supplementary material for: Multiplex real-time reverse transcription recombinase-aided amplification assay for the detection of SARS-CoV-2, influenza A virus, and respiratory syncytial virus
Source: Microbiol Spectr. 2025 May 5;13(6):e02759-24. doi: 10.1128/spectrum.02759-24 (PMC12131773; doi:10.1128/spectrum.02759-24)
Supplement: Supplemental figures — Fig. S1 and S2. [file spectrum.02759-24-s0001.docx]

**Supplement figures and legends**

#
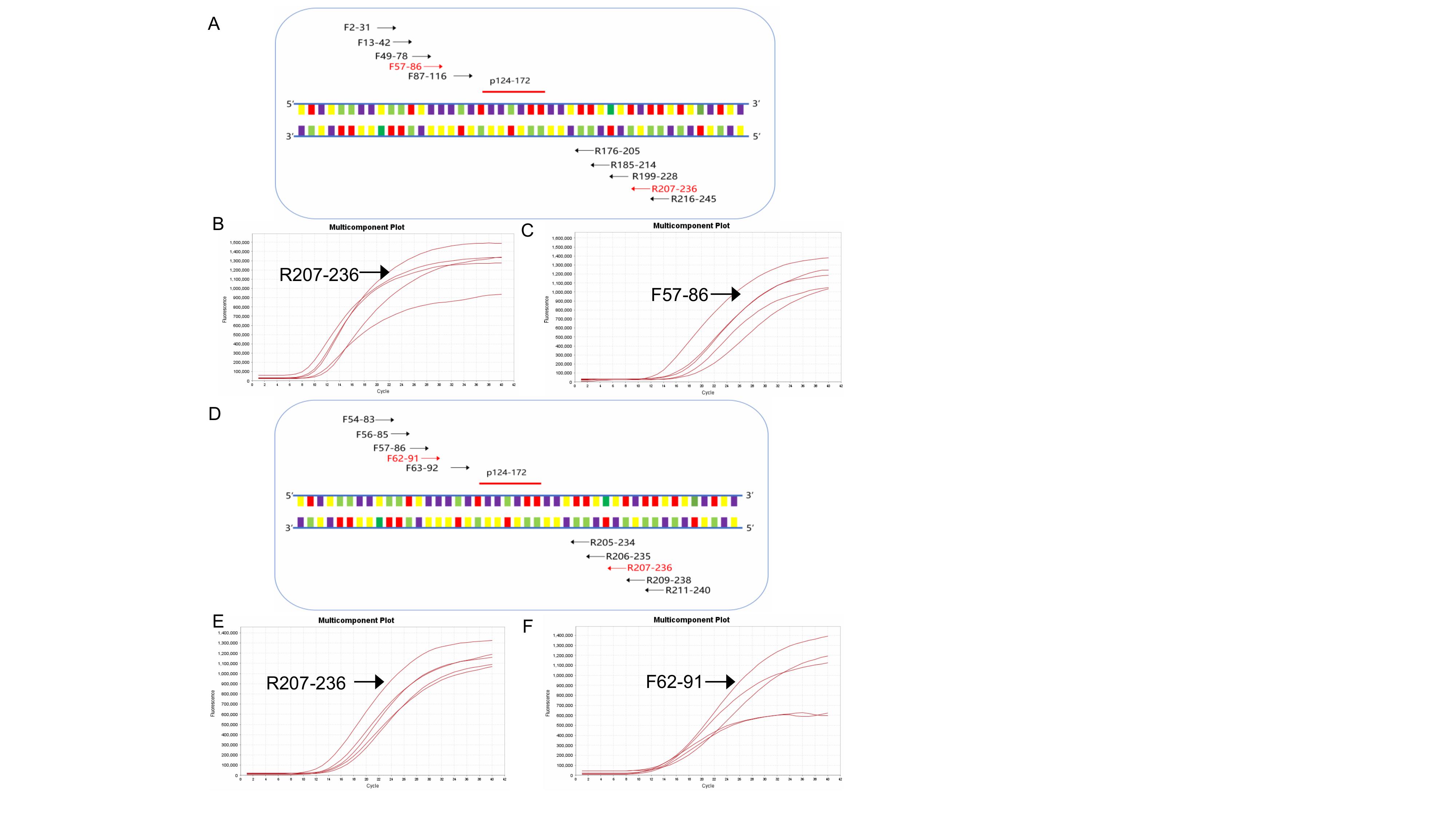


# Figure S1 Screening of optical primers for real-time RT-RAA detection of IAV.

**(A**) Schematic representation of the primary primer screening process. In the primer nomenclature, the numerical values denote the specific positions within the M gene of IAV (GenBank accession no. MN570352.1). **(B)** Results of the primary reverse primer screening. The forward primer F2-31 was randomly selected to evaluate all five reverse primers. **(C)** Results of the primary forward primer screening. The selected reverse primer R202-231 was utilized to assess all five forward primers. **(D)** Schematic representation of the secondary primer screening process. **(E)** Results of the secondary reverse primer screening. The selected forward primer F87-116 was employed to evaluate all seven reverse primers. **(F)** Results of the secondary forward primer screening. The selected reverse primer R197-230 was utilized to assess all seven forward primers.


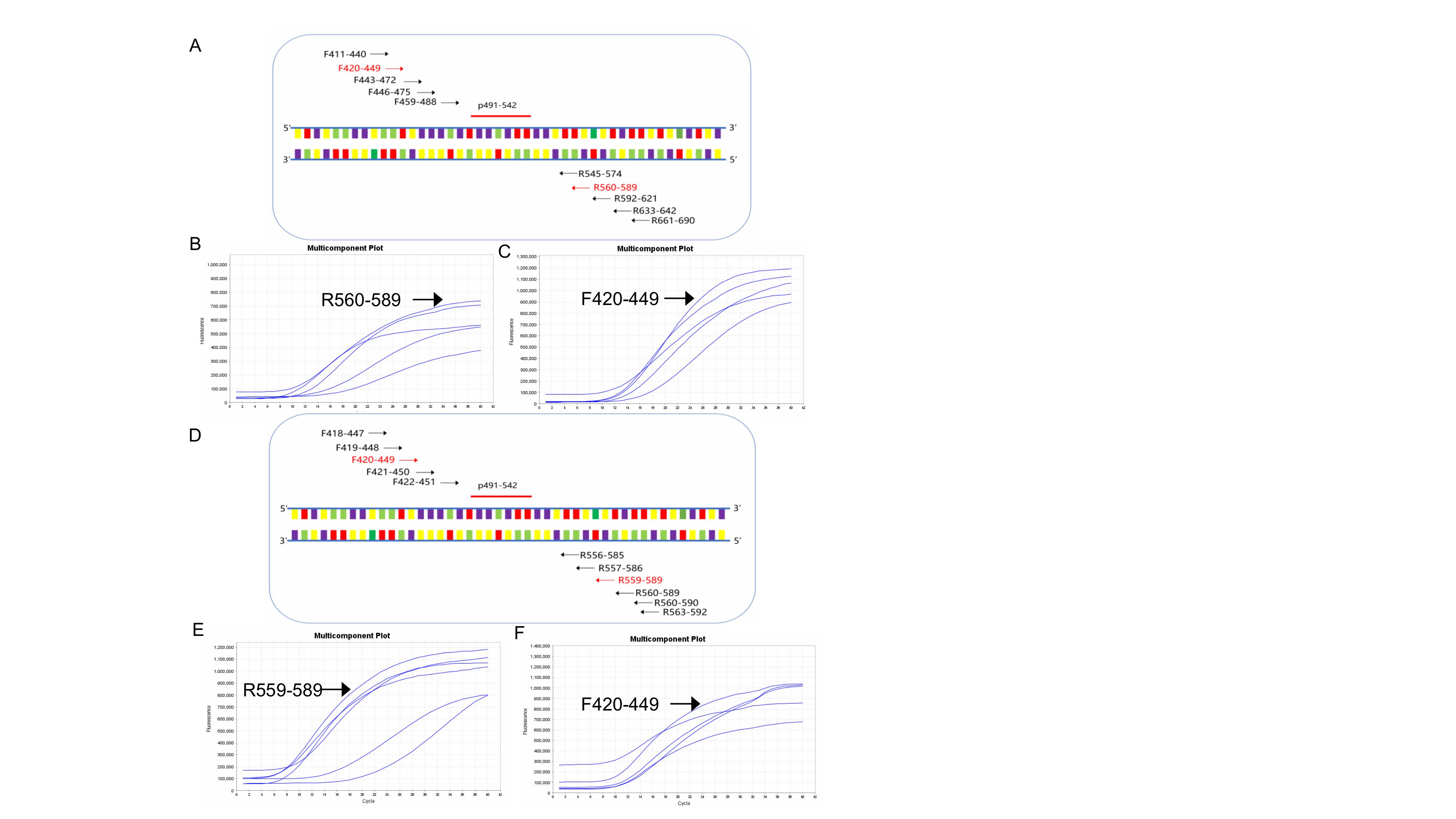


# Figure S2 Screening of optical primers for real-time RT-RAA detection of RSV.

**(A)** Schematic representation of the primary primer screening process. In the primer nomenclature, the numerical values denote the specific positions within the N gene of RSV (GenBank accession no. MN310477.1). **(B)** Results of the primary reverse primer screening. The forward primer F443-472 was randomly selected to evaluate all five reverse primers. **(C)** Results of the primary forward primer screening. The selected reverse primer R560-589 was utilized to assess all five forward primers. **(D)** Schematic representation of the secondary primer screening process. **(E)** Results of the secondary reverse primer screening. The selected forward primer F420-449 was employed to evaluate all seven reverse primers. **(F)** Results of the secondary forward primer screening. The selected reverse primer R559-589 was utilized to assess all seven forward primers.
