# Supplementary material for: Multiplex real-time reverse transcription recombinase-aided amplification assay for the detection of SARS-CoV-2, influenza A virus, and respiratory syncytial virus
Source: Microbiol Spectr. 2025 May 5;13(6):e02759-24. doi: 10.1128/spectrum.02759-24 (PMC12131773; doi:10.1128/spectrum.02759-24)
Supplement: Table S1 — Specific information on clinical samples. [file spectrum.02759-24-s0002.docx]

Supplementary Table

# Table S1 Specific information on clinical samples

| **Sample**  **number** | **Sample**  **type** | **IAV**  **Ct value** | **SARS-CoV-2**  **Ct value** | **RSV**  **Ct value** | **Triple**  **RT-qPCR** | **Triple real-**  **time RT-RAA** |
| --- | --- | --- | --- | --- | --- | --- |
| A3 | Cultured isolates | 20.122 | - | - | IAV | IAV |
| A4 | Cultured isolates | 30.630 | - | - | IAV | IAV |
| B6 | Cultured isolates | 23.460 | - | - | IAV | IAV |
| B18 | Cultured isolates | 23.540 | - | - | IAV | IAV |
| C2 | Cultured isolates | 19.935 | - | - | IAV | IAV |
| C4 | Cultured isolates | 21.578 | - | - | IAV | IAV |
| C6 | Cultured isolates | 20.826 | - | - | IAV | IAV |
| C31 | Cultured isolates | 20.540 | - | - | IAV | IAV |
| C32 | Cultured isolates | 21.509 | - | - | IAV | IAV |
| C33 | Cultured isolates | 19.863 | - | - | IAV | IAV |
| C34 | Cultured isolates | 23.962 | - | - | IAV | IAV |
| D5 | Cultured isolates | 18.167 | - | - | IAV | IAV |
| D6 | Cultured isolates | 21.005 | - | - | IAV | IAV |
| D10 | Cultured isolates | 20.578 | - | - | IAV | IAV |
| D13 | Oropharyngeal swabs | 33.479 | - | - | IAV | IAV |
| D32 | Cultured isolates | 19.886 | - | - | IAV | IAV |
| Z1 | Oropharyngeal swabs | 29.519 | - | - | IAV | IAV |
| E6 | Oropharyngeal swabs | 29.052 | - | - | IAV | IAV |
| SD1 | Oropharyngeal swabs | - | 31.147 | - | SARS-CoV-2 | SARS-CoV-2 |
| SD2 | Oropharyngeal swabs | - | 38.115 | - | SARS-CoV-2 | SARS-CoV-2 |
| SD3 | Oropharyngeal swabs | - | 31.117 | - | SARS-CoV-2 | SARS-CoV-2 |
| SD4 | Oropharyngeal swabs | - | 26.631 | - | SARS-CoV-2 | SARS-CoV-2 |
| SD5 | Oropharyngeal swabs | - | 31.151 | - | SARS-CoV-2 | SARS-CoV-2 |
| SD6 | Oropharyngeal swabs | - | 28.595 | - | SARS-CoV-2 | SARS-CoV-2 |
| SD7 | Oropharyngeal swabs | - | 27.889 | - | SARS-CoV-2 | SARS-CoV-2 |
| SD8 | Oropharyngeal swabs | - | 26.856 | - | SARS-CoV-2 | SARS-CoV-2 |
| SD9 | Oropharyngeal swabs | - | 32.409 | - | SARS-CoV-2 | SARS-CoV-2 |
| SD10 | Oropharyngeal swabs | - | 26.279 | - | SARS-CoV-2 | SARS-CoV-2 |
| JC1098 | Oropharyngeal swabs | - | 32.349 | - | SARS-CoV-2 | SARS-CoV-2 |
| JC1112 | Oropharyngeal swabs | - | - | - | - | - |
| JC1131 | Oropharyngeal swabs | - | - | - | SARS-CoV-2 | SARS-CoV-2 |
| JC1134 | Oropharyngeal swabs | - | 18.774 | - | SARS-CoV-2 | SARS-CoV-2 |
| JC1141 | Oropharyngeal swabs | - | 33.928 | - | SARS-CoV-2 | SARS-CoV-2 |
| JC1162 | Oropharyngeal swabs | - | 33.715 | - | SARS-CoV-2 | SARS-CoV-2 |
| JC1172 | Oropharyngeal swabs | - | - | - | - | - |
| JC1181 | Oropharyngeal swabs | - | 26.465 | - | SARS-CoV-2 | SARS-CoV-2 |
| JC1186 | Oropharyngeal swabs | - | 29.197 | - | SARS-CoV-2 | SARS-CoV-2 |
| JC1207 | Oropharyngeal swabs | - | - | - | - | - |
| JC1202 | Oropharyngeal swabs | - | 30.542 | - | SARS-CoV-2 | SARS-CoV-2 |
| JC1204 | Oropharyngeal swabs | - | 19.726 | - | SARS-CoV-2 | SARS-CoV-2 |
| JC1221 | Oropharyngeal swabs | - | 24.910 | - | SARS-CoV-2 | SARS-CoV-2 |
| JC1222 | Oropharyngeal swabs | - | 31.413 | - | SARS-CoV-2 | SARS-CoV-2 |
| JC1238 | Oropharyngeal swabs | - | 26.617 | - | SARS-CoV-2 | SARS-CoV-2 |
| JC1243 | Oropharyngeal swabs | - | 12.759 | - | SARS-CoV-2 | SARS-CoV-2 |
| JC1252 | Oropharyngeal swabs | - | 31.007 | - | SARS-CoV-2 | SARS-CoV-2 |
| JC1275 | Oropharyngeal swabs | - | 30.627 | - | SARS-CoV-2 | SARS-CoV-2 |
| JC1283 | Oropharyngeal swabs | - | 22.328 | - | SARS-CoV-2 | SARS-CoV-2 |
| JC1293 | Oropharyngeal swabs | - | 28.214 | - | SARS-CoV-2 | SARS-CoV-2 |
| E27 | Oropharyngeal swabs | - | 29.681 | - | SARS-CoV-2 | SARS-CoV-2 |
| E31 | Oropharyngeal swabs | - | 30.940 | - | SARS-CoV-2 | SARS-CoV-2 |
| G1 | Alveolar lavage fluid | - | - | 31.423 | RSV | RSV |
| G10 | Alveolar lavage fluid | - | - | 34.398 | RSV | RSV |
| SD556 | Oropharyngeal swabs | - | - | - | - | - |
| SD657 | Oropharyngeal swabs | - | - | 29.587 | RSV | RSV |
| SD948 | Oropharyngeal swabs | - | - | - | - | - |
| SD965 | Oropharyngeal swabs | - | - | 35.075 | RSV | RSV |
| O2SD458 | Oropharyngeal swabs | - | - | 31.458 | RSV | RSV |
| O2SD461 | Oropharyngeal swabs | - | - | 31.386 | RSV | RSV |
